# Inhibition of Hepatitis B Virus Replication by Helper Dependent Adenoviral Vectors Expressing Artificial Anti-HBV Pri-miRs from a Liver-Specific Promoter

**DOI:** 10.1155/2014/718743

**Published:** 2014-06-05

**Authors:** Mohube Betty Mowa, Carol Crowther, Abdullah Ely, Patrick Arbuthnot

**Affiliations:** Antiviral Gene Therapy Research Unit, School of Pathology, Health Sciences Faculty, University of the Witwatersrand, Private Bag 3, Johannesburg 2050, South Africa

## Abstract

Research on applying RNA interference (RNAi) to counter HBV replication has led to identification of potential therapeutic sequences. However, before clinical application liver-specific expression and efficient delivery of these sequences remain an important objective. We recently reported short-term inhibition of HBV replication *in vivo* by using helper dependent adenoviral vectors (HD Ads) expressing anti-HBV sequences from a constitutively active cytomegalovirus (CMV) promoter. To develop the use of liver-specific transcription regulatory elements we investigated the utility of the murine transthyretin (MTTR) promoter for expression of anti-HBV primary microRNAs (pri-miRs). HD Ads containing MTTR promoter effected superior expression of anti-HBV pri-miRs in mice compared to HD Ads containing the CMV promoter. MTTR-containing HD Ads resulted in HBV replication knockdown of up to 94% in mice. HD Ads expressing trimeric anti-HBV pri-miRs silenced HBV replication for 5 weeks. We previously showed that the product of the codelivered *lacZ* gene induces an immune response, and the duration of HBV silencing *in vivo* is likely to be attenuated by this effect. Nevertheless, expression of anti-HBV pri-miRs from MTTR promoter is well suited to countering HBV replication and development of HD Ads through attenuation of their immunostimulatory effects should advance their clinical utility.

## 1. Introduction

Reactivation of hepatitis B virus (HBV) replication after treatment withdrawal commonly occurs with currently available anti-HBV therapies [1, 2]. This has necessitated development of more effective durable approaches to countering HBV infection. RNA interference- (RNAi-) based therapy has shown promising outcomes as an alternative to current hepatitis B treatment (reviewed in [3, 4]). Naturally, the RNAi pathway uses small RNAs (e.g., microRNAs (miRs)) to regulate gene expression in a wide range of organisms. Binding of small RNAs to the target sequence may result in degradation or translation inhibition of the target. Generally, endogenous activation of the RNAi pathway with miRs initially involves transcription of primary miRs (pri-miRs) by RNA polymerase (Pol) II. These hairpin-like structures are then processed in the nucleus by Drosha (RNase III) and its double stranded RNA binding partner DGCR8, to form precursor miRs (pre-miRs). Pre-miRs are then exported to the cytoplasm and processed by Dicer (RNase III) to form mature miR duplexes. Mature miRs are passed on to the RNA induced silencing complex (RISC) containing the argonaute protein with RNase activity. One strand is selected as a guide, which pairs with the target to facilitate target translation inhibition or degradation (reviewed in [5]).

Manipulation of RNAi for therapeutic purposes involves the use of synthetic short interfering RNA (siRNA), expressed pri-miR or pre-miR mimics. These artificial intermediates of the RNAi pathway reprogramme the endogenous RNAi pathway to induce silencing of specific targets (reviewed in [6]). Expressed RNAi activators have advantages of sustained efficacy from continuous intracellular supply of siRNAs, ease of propagation in plasmid DNA, better stability, and compatibility with highly efficient viral vectors. These properties make them well suited to treatment of chronic viral infections, such as caused by HBV. Pol III promoters, for example, U6 small nuclear RNA (snRNA) and human ribonuclease P RNA component H1 promoters, are commonly used to regulate transcription of short hairpin RNAs (shRNAs) that mimic pre-miRs [7, 8]. However, overexpression from Pol III promoters may be complicated by toxicity that is attributed to saturation of the endogenous RNAi machinery [9, 10]. The versatility of Pol II promoters overcomes some of these problems. These transcriptional regulatory elements are capable of achieving tissue specific transgene expression, improved transcriptional regulation, and generation of multimeric pri-miR activators of RNAi [11, 12]. Since functional coupling between intermediates of the RNAi pathway may occur, expression of earlier intermediates of the RNAi pathway, such as pri-miRs, may improve efficiency of target silencing. Moreover, use of artificial pri-miR cassettes that are expressed from Pol II promoters has been shown to be safer than employing U6 Pol III shRNA expression cassettes [11, 13–15]. Use of a liver-specific promoter to express anti-HBV sequences is also potentially useful to limit nonspecific effects that may be caused by constitutively active transcriptional regulatory elements.

Achieving safe and efficient nucleic acid delivery is important for developing RNAi-based therapy of viral infections. The natural targeting of hepatocytes by adenoviral vectors [16] and sustained transgene expression that may be attained with helper dependent adenoviral vectors (HD Ads) makes them suitable for delivering expressed RNAi activators that are designed to treat chronic HBV infection (reviewed in [17, 18]). The feasibility of using HD Ads for treating HBV infection has been demonstrated in murine and woodchuck models of the disease [19–22]. In these studies, immunotherapy-based treatment using HD Ads to deliver interferon-alpha and interleukin-12 was employed. Prolonged (~3 months) transgene expression with sustained antiviral effects was observed with HD Ads, which was not possible with first generation Ad vector expressing interferon-alpha.

Rauschhuber et al. reported the efficacy of HD Ad expressing shRNAs against HBV infection in mice [23]. However, modest silencing of HBV replication was observed and is likely to be a result of modest silencing efficacy of the antiviral shRNA expression cassettes. Using a HBV transgenic mouse model of chronic HBV infection, we recently reported high efficacy of transduction and superior silencing of HBV replication by HD Ads expressing previously designed artificial antiviral pri-miR and pre-miR mimics [11, 13]. Constitutively active U6 Pol III and cytomegalovirus (CMV) Pol II promoters were used effectively to inhibit HBV replication with pre-miR and pri-miR mimics, respectively [24, 25]. In the case of the artificial HBV-targeting pri-miRs, natural miR-122 and miR-31 sequences were used as templates to design the exogenous RNAi activators. Highly efficient inhibition of HBV replication was observed. To improve this approach we have incorporated a liver-specific modified mouse transthyretin (MTTR) promoter [26] into the pri-miR expression cassettes. Use of HD Ads to deliver these antiviral sequences to hepatocytes* in vivo* resulted in effective gene silencing without evidence of off target effects. Moreover, in vivo generation of processed mature miR sequences from MTTR-containing cassettes was more efficient when compared to that achieved with the CMV promoter. Although liver-specific promoters have previously been employed to express anti-HBV RNAi activators with varying success [12, 27], use of the well characterised and highly efficient liver-specific MTTR promoter sequence has not been investigated. Demonstration here that this transcriptional regulatory element may be used to work efficiently against HBV in vivo indicates that the MTTR promoter has utility for therapeutic hepatic transgene expression.

## 2. Materials and Methods

### 2.1. Construction of Adenoviral Genome Bearing Plasmids

Generation of adenoviral plasmids expressing anti-HBV pri-miRs from CMV promoter has previously been described [25]. To generate adenoviral plasmids expressing anti-HBV pri-miRs from the MTTR promoter, a CMV promoter sequence in a previously described pCI-neo plasmid containing anti-HBV single or trimeric pri-miR sequences (pri-miR-122/5, pri-miR-31/5 and pri-miR-31/5-8-9) [11] was substituted with MTTR promoter sequence [26] using* Bgl *II and* Sac* I restriction sites. This generated pMTTR pri-miR-122/5, pMTTR pri-miR-31/5, and pMTTR pri-miR-31/5-8-9 plasmids. The MTTR promoter-pri-miR cassettes were then amplified using PCR with a previously described primer set (5′ GAT CGG CGC GCC CTA TGG AAA AAC GCC AGC AA 3′ and 5′ GAT CGG CGC GCC GAA AGG AAG GGA AGA AAG CGA 3′) that incorporated flanking* Asc* I restriction sites (underlined) in the amplicons [25]. Amplified fragments were then inserted into pTZ57R/T (InsTAclone PCR cloning Kit, Fermentas, MD, USA) to generate pTZMTTR-pri-miR-122/5, pTZMTTR-pri-miR-31/5, and pTZMTTR-pri-miR-31/5-8-9. Following sequencing, the inserts were removed using* Asc* I and subcloned at the* Asc* I restriction site of pΔ28E4LacZ adenoviral backbone [28] to generate pΔ28E4LacZ MTTR pri-miR-122/5, pΔ28E4LacZ MTTR pri-miR-31/5, and pΔ28E4LacZ MTTR pri-miR-31/5-8-9.

### 2.2. Luciferase Assay to Determine Liver-Specific Expression of MTTR Promoter

In vitro hepatotropic expression of luciferase from MTTR promoter was determined using the Dual-Luciferase Reporter Assay System according to manufacturer's instructions (Promega, WI, USA). Huh7 or HEK293 cells were cotransfected with 100 ng of pMTTR-FLuc or pCMV-FLuc [29] and 100 ng of phRL-CMV (Promega, WI, USA) plasmids. Forty-eight hours after transfection, cells were harvested and lysed. The cell lysates were used for luciferase activity measurement. Firefly luciferase expression was normalised to* Renilla* luciferase expression.

### 2.3. Production of Anti-HBV HD Ads

Production, amplification, and purification of anti-HBV HD Ads were carried out according to published methods [28]. Briefly, HEK293-derived 116 HD Ad packaging cell line was propagated in Eagle's or Joklik's minimum essential medium (MEM). Following transfection of 116 cells with HD Ad genome-containing plasmid DNA that had been linearized by* Pme* I restriction digestion, cells were infected with helper virus (AdNG163). After 48 hours of incubation, the cells were harvested and frozen at −80°C. Transfected cell suspensions were subjected to three cycles of freeze-thawing and the lysates then used to coinfect 116 cells with helper virus, and incubated for 48 hours. This was repeated several times until viral titers were maximal. For large scale preparation of anti-HBV HD Ads, 2 liter cultures of 116 cells were coinfected with helper virus and anti-HBV HD Ad lysates. Two days following coinfection, cells were harvested by centrifugation, the cells were then lysed and anti-HBV HD Ads were purified using CsCl gradient centrifugation. Total viral particles and helper virus contamination were determined using quantitative PCR (Q-PCR).

To determine viral infectious units, HEK293 cells infected with serial dilutions of HD Ads were stained for *β*-galactosidase activity as previously described [30]. Preparations of viral particles were aliquoted and stored in 10% glycerol at −80°C. The complete panel of HD Ads used in this study therefore comprised the previously described HD Ad Δ28, HD Ad HBV CMV pri-miR-31/5, HD Ad HBV CMV pri-miR-122/5, HD Ad HBV CMV pri-miR-31/5-8-9, and newly propagated HD Ad HBV MTTR pri-miR-31/5, HD Ad HBV MTTR pri-miR-122/5, and HD Ad HBV MTTR pri-miR-31/5-8-9, which express antiviral sequences from the MTTR promoter.

### 2.4. Assessment of Efficacy in Cultured Cell of Anti-HBV HD Ads

To assess anti-HBV effects in vitro, the liver-derived Huh7 cell line was used and maintained as previously described [30]. Huh7 cells were cotransfected with a target plasmid vector containing a greater than genome length HBV sequence (pCH-9/3091) [31] and a plasmid expressing enhanced green fluorescent protein (eGFP) [32] as a transfection control. Lipofectamine 2000 was used for all transfections according to manufacturer's instructions (Life Technologies, CA, USA). Five hours after transfection, cells were washed with fresh medium and infected with HD Ads at multiplicities of infection (MOIs) ranging from 100 to 1000 infectious viral particles per cell. At 48 hours after HD Ad infection, secretion of HBV surface antigen (HBsAg) into the culture supernatants was measured as a marker of HBV replication using MONOLISA HBs Ag Assay kit (Bio-Rad, CA, USA).

### 2.5. Northern Blot Hybridization

To determine pri-miR expression and processing in cell culture, Huh7 cells were infected with HD Ads at a MOI of 100 infectious viral particles per cell. Two days after infection, cells were harvested and total RNA was extracted using Tri Reagent (Sigma, MI, USA). To determine pri-miR expression and processing in vivo, HBV transgenic mice [33] were used according to the protocols approved by the University of the Witwatersrand Animal Ethics Screening Committee. Mice were injected with a dose of 5 × 10^9^ HD Ad infectious viral particles via the tail vein. Animals were then killed at 1 week after injection and livers were harvested. Livers were homogenized and total RNA was isolated using Tri Reagent (Sigma, MI, USA). Thirty or sixty micrograms of RNA from cultured cells or liver tissue were analyzed using Northern blot hybridization according to previously described methods [11, 13]. Labelled probes with sequences of 5′ CCG TGT GCA CTT CGC TTC 3′, 5′ CAA TGT CAA CGA CCG ACC 3′, and 5′ TAG GAG GCT GTA GGC ATA 3′ were used to detect mature guides 5, 8, and 9, respectively. Blots were then stripped and probed for U6 snRNA using a labelled oligonucleotide with sequence 5′ TAG TAT ATG TGC TGC CGA AGC GAG CA 3′. Band intensities were quantified by measuring photostimulated luminescence (PSL) using a FUJI FILM phosphorimager with Multi Gauge software.

### 2.6. Administration of Anti-HBV HD Ads to HBV Transgenic Mice

HBV transgenic mice [33] were used as a model of HBV replication, and protocols employed had been approved by the University of the Witwatersrand Animal Ethics Screening Committee. Six mice per group were infected by injecting a single dose of 5 × 10^9^ infectious viral particles via the tail vein. Transduction of HD Ads and* lacZ* gene expression was assessed by X-gal staining of frozen liver sections. Frozen sections were prepared according to standard protocol. Blood samples were collected over a period of 13 weeks by retroorbital puncture. Serum HBsAg was measured by ELISA using the MONOLISA HBs Ag ULTRA kit (Bio-Rad, CA, USA). Circulating viral particle equivalents (VPEs) were determined by real-time Q-PCR according to previously described methods [13, 34].

To determine hepatotoxicity of the anti-HBV HD Ads, six mice per group were injected with 5 × 10^9^ infectious viral particles via the tail vein. Blood samples were taken after infection and alanine transaminase (ALT) activity was determined in the serum.

### 2.7. Statistical Analysis

Data are expressed as the mean ± standard error of the mean (SEM). Statistical difference was determined using Student's 2-tailed *t*-test and was considered significant when *P* ≤ 0.05. Calculations were done with the GraphPad Prism software (GraphPad Software Inc., CA, USA).

## 3. Results

### 3.1. Structure of HD Ads Containing CMV and MTTR Pol II Artificial Anti-HBV Pri-miR Expression Cassettes

**Figure 1:**
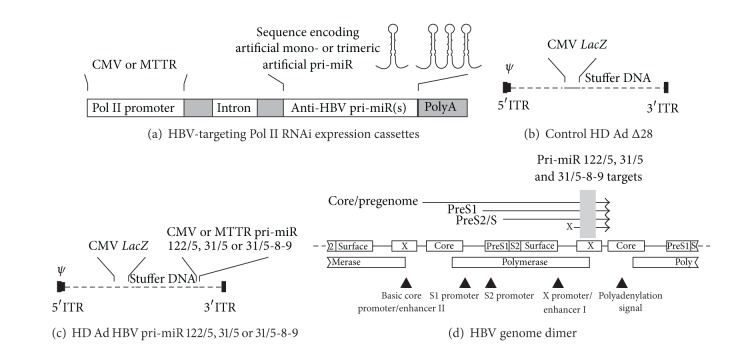
Schematic illustration of artificial pri-miR expression cassettes, recombinant HD Ads and HBV target sites. (a) Mono- or trimeric anti-HBV pri-miR sequences are transcribed under control of either a CMV or MTTR promoter. The cassettes also include an intron and polyA transcription termination signal. (b) The control HD Ad Δ28 vector contains no HBV-targeting cassettes but does include the CMV* LacZ* reporter cassette. Apart from ITRs and viral packaging signal (Ψ) the remainder of the vector DNA comprises stuffer sequence with all Ad open reading frames (ORFs) removed. (c) Anti-HBV HD Ads contain the mono- or trimeric artificial pri-miR expression cassettes under control of the CMV or MTTR Pol II promoters. (d) Tandem arrangement of a dimer of the hepatitis B virus genome, comprising 6.4 kb, showing sites targeted by antiviral artificial pri-miRs. The overlapping* surface*,* core*,* polymerase,* and* HBx* viral ORFs are indicated by labelled rectangles. The four arrows above the genome represent the HBV transcripts that are initiated from different viral promoter elements and a common 3′ end. Essential cis elements controlling transcription are indicated by arrowheads. The sites targeted by the artificial pri-miRs, indicated by the shaded rectangle, are located within the* HBx* ORF and are found in all of the viral transcript.

Previously described mono- and trimeric anti-HBV pri-miR sequences were inserted downstream of the constitutively active CMV or liver-specific MTTR Pol II promoter sequences (Figure 1(a)) [11]. Artificial monomeric pri-miR sequences, pri-miR-122/5, pri-miR-31/5, used the pri-miR-122 or pri-miR-31 sequences as a scaffold for incorporating a HBV-targeting guide sequence. The pri-miR-31/5-8-9 sequence was incorporated into trimeric cassettes and comprised a tandem arrangement of three different HBV-targeting pri-miR-31 derivatives. These cassettes had previously been shown to produce transcripts that were efficiently processed and capable of silencing HBV replication in cultured liver cells and in vivo. Expression cassettes were incorporated into HD Ads to generate a panel of six vectors that each expressed pri-miR-122/5, pri-miR-31/5, or pri-miR-31/5-8-9 from either the CMV or MTTR Pol II promoters (Figure 1(b)). A control vector, HD Ad Δ28, did not contain any anti HBV sequence. All HD Ads also included a reporter cassette encoding Beta-galactosidase to enable convenient tracking of cells infected by the viral vectors. The targets of the guides generated from the artificial expressed RNAi activators are located in the* HBx* open reading frame (ORF) of HBV (Figure 1(c)) [30]. This viral sequence is conserved in HBV genotypes and is also present in all of the viral transcripts. The replication-competent integrant that had been inserted into the transgenic mice used as a model of viral replication in this study comprised a dimer of HBV sequences (Figure 1(c)) [33].

### 3.2. Inhibition of HBV Replication by HD Ads Carrying CMV Promoter Driven Anti-HBV Sequences in Mice

**Figure 2:**
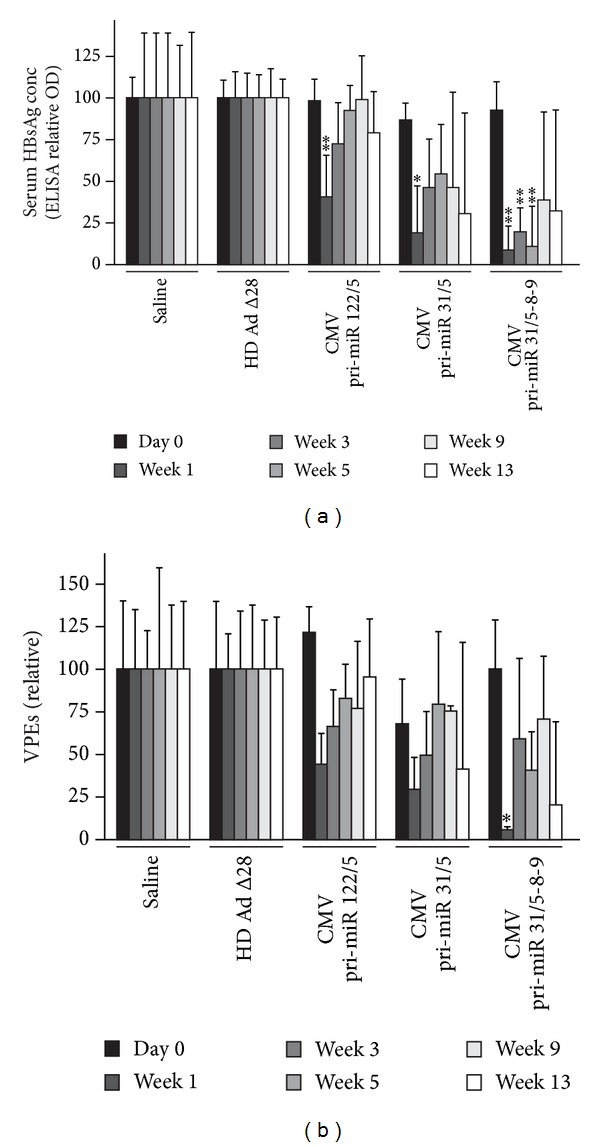
Silencing of HBV replication in mice by HD Ads expressing anti-HBV pri-miR mimics from a CMV promoter. Mice were infected with a single dose of 5 × 10^9^infectious HD Ad particles expressing pri-miR-122/5, pri-miR-31/5, and pri-miR-31/5-8-9. Animals injected with saline or HD Ad Δ28 served as negative controls. (a) Time course of serum HBsAg concentrations, as measured by ELISA, following HD Ad administration. (b) Circulating viral particle equivalents (VPEs) were measured using qPCR following injection of HBV transgenic mice with HD Ads. Data are expressed as means with SEMs from each group of six mice. The statistical significance was calculated using a pair-wise comparison according to the Student *t*-test. *P* values less than 0.05 (∗) or less than 0.01 (∗∗) were considered statistically significant.

We previously reported a significant decrease in serum HBsAg levels and VPEs at one and two weeks after treatment of HBV transgenic mice with HD Ads expressing anti-HBV pri-miR mimics from a CMV promoter [25]. To assess long-term anti-HBV effect in mice, six mice per group were injected with HD Ads carrying CMV promoter-anti-HBV pri-miR cassettes and monitored over a 13-week period. In agreement with the previous report, significant decrease in HBsAg levels was observed at one week following the injection of mice with either HD Ad HBV pri-miR-122/5, HD Ad HBV pri-miR-31/5, or HD Ad HBV pri-miR-31/5-8-9 (Figure 2(a)). In mice treated with HD Ad HBV pri-miR-122/5 or HD Ad HBV pri-miR-31/5, the serum concentrations of HBsAg increased at time points after one week and were no longer significantly different from the baseline values. However, HD Ad HBV pri-miR-31/5-8-9 administration resulted in the most efficient silencing of HBsAg expression. Inhibition of up to 94% was achieved at 1 week and prolonged anti-HBV effects were observed for five weeks following administration of HD Ad HBV pri-miR-31/5-8-9. Similar, although less statistically significant, results were observed when VPEs were measured as a marker of HBV replication (Figure 2(b)).

### 3.3. Expression of Anti-HBV Sequences from MTTR Promoter and HBV Replication Inhibition in Cell Culture

**Figure 3:**
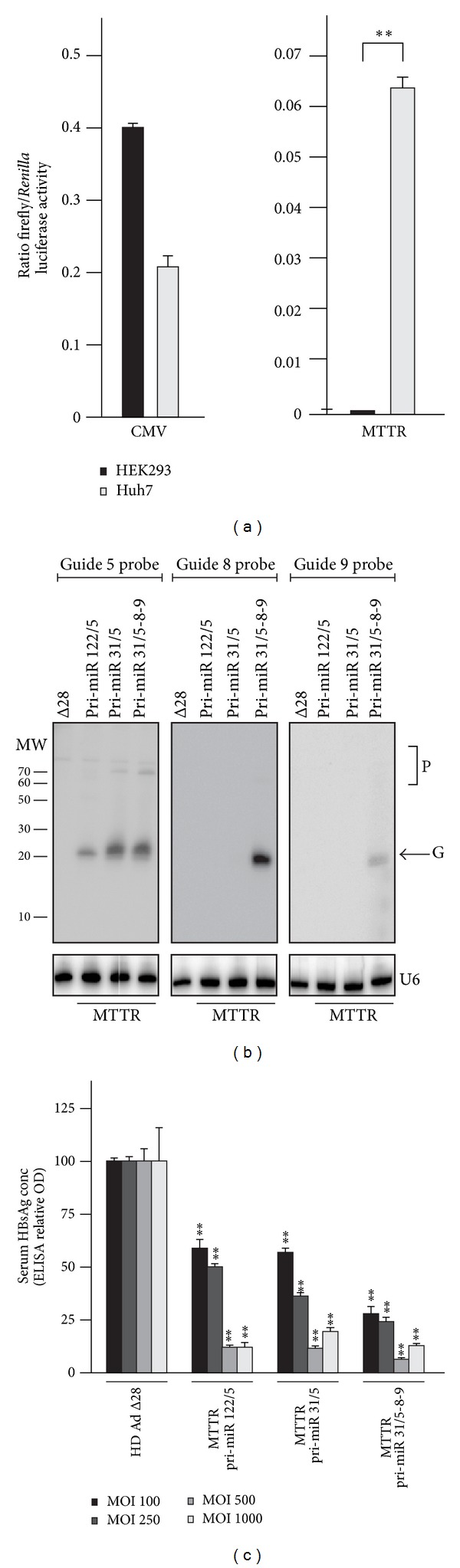
Liver specificity of MTTR promoter, RNA processed from anti-HBV pri-miR cassettes, and effect on HBsAg concentration in supernatants of cultured Huh7 cells. (a) Liver-derived Huh7 or kidney-derived HEK293 cells were transfected with 100 ng of pMTTR-FLuc or pCMV-FLuc, which contained the* Firefly luciferase* reporter gene under control of MTTR or CMV promoters, respectively. In addition the phRL-CMV (Promega) plasmid, constitutively expressing* Renilla* luciferase, was cotransfected to normalize the Firefly luciferase measurements. Forty-eight hours after transfection cells were harvested and activity of the two reporter genes was determined independently. Mean Firefly to* Renilla* luciferase activity is presented and error bars indicate the standard error of the mean (*n* = 4). (b) Northern blot analyses of 30 *μ*g RNA isolated from Huh 7 cells infected with HD Ads at a multiplicity of infection (MOI) of 100 infectious viral particles per cell. The probes used were complementary to the predicted 5, 8, or 9 guide sequences derived from the panel of vectors expressing antiviral pri-miRs from the MTTR promoter. Equal loading of the lanes was verified by stripping and reprobing the blot with a labelled oligonucleotide complementary to U6 small nuclear RNA. Putative guide (G) and precursor (P) bands are indicated. (c) Inhibition of HBV replication in vitro was determined by measuring HBsAg levels using ELISA. Cell culture supernatants were obtained from Huh7 cells that had been transfected with the pCH-9/3091 replication-competent plasmid and then infected with the indicated HD Ads at MOIs of 100, 250, 500, or 1000. Data is expressed as SEM from three replicates. The statistical significance was calculated using a pair-wise comparison according to the Student* t*-test. *P* values less than 0.05 (∗) or less than 0.01 (∗∗) were considered statistically significant.

Despite the high efficacy and prolonged knockdown of HBV replication by HD Ad HBV pri-miR-31/5-8-9, previous studies have shown that hepatic transgene expression from the CMV promoter is not durable [35, 36]. To address this limitation, HD Ad vectors expressing anti-HBV pri-miRs from a liver-specific MTTR promoter were generated. Initially, liver-specific expression of the MTTR promoter was assessed using a reporter gene assay following transfection of liver-derived (Huh7) and kidney-derived (HEK293) cells (Figure 3(a)). Measurement of Firefly luciferase activity showed reporter gene activity in both cell lines receiving the plasmid containing the constitutively active CMV promoter. However, when cells were transfected with cassettes containing the MTTR transcriptional regulatory element, Firefly luciferase activity was barely detectable in the kidney derived cell line, and activity was significantly higher in the liver-derived cell line. To assess processing of the artificial pri-miRs, RNA isolated from Huh7 cells infected with the MTTR-containing HD Ads at a MOI of 100 infectious viral particles per cell were analyzed by low molecular weight northern blot hybridization (Figure 3(b)). Hybridization using a probe complementary to guide 5 confirmed efficient generation of anti-HBV pri-miRs from each of the MTTR promoter-containing monomeric and trimeric HD Ads. This was evident from the detection of a band of approximately 21 nucleotides in length, which was absent from control cells infected with the empty HD Ad Δ28 vector. Higher molecular weight bands representing unprocessed or partially processed intermediates were present in low amounts. As expected, hybridization to a probe complementary to the guide 8 sequence resulted in detection of a putative guide in RNA samples isolated from cells infected with HD Ad HBV pri-miR-31/5-8-9 but not in HD Ad HBV pri-miR-122/5 or HD Ad HBV pri-miR-31/5. Similarly, the probe detecting guide 9 sequences only detected a band in RNA isolated from cells treated with the trimeric HD Ad vector. The lower concentration of the guide 9 sequence is in accordance with our previous observation that this sequence is the least efficiently processed guide derived from the artificial tricistronic cassette [11]. A band of approximately 21 nucleotides was not detected in RNA samples isolated from cells infected with empty vector (HD Ad Δ28) or uninfected cells (Figure 3(b)).

To assess HBV silencing efficacy of MTTR promoter-containing HD Ads in cell culture, HBsAg concentrations in culture supernatants were measured as a marker for HBV replication. Huh7 cells were initially transfected with the pCH-9/3091 HBV replication competent target plasmid [31]. Five hours after transfection, cells were infected with HD Ads at MOIs ranging from 100 to 1000 infectious viral particles per cell. HBsAg levels were measured 48 hours after infection. All three anti-HBV HD Ads (HD Ad HBV pri-miR-31/5-8-9 M, HD Ad HBV pri-miR-31/5 M, and HD Ad HBV pri-miR-122/5 M) resulted in a significant dose dependent decrease in HBsAg levels. As expected most efficient inhibition of this HBV replication marker was observed at MOIs of 500 and 1000 infectious viral particles per cell, with knockdown of up to 91% (Figure 3(c)).

### 3.4. Comparison of Expression and Processing In Vivo of CMV- and MTTR-Derived Anti-HBV Sequences

**Figure 4:**
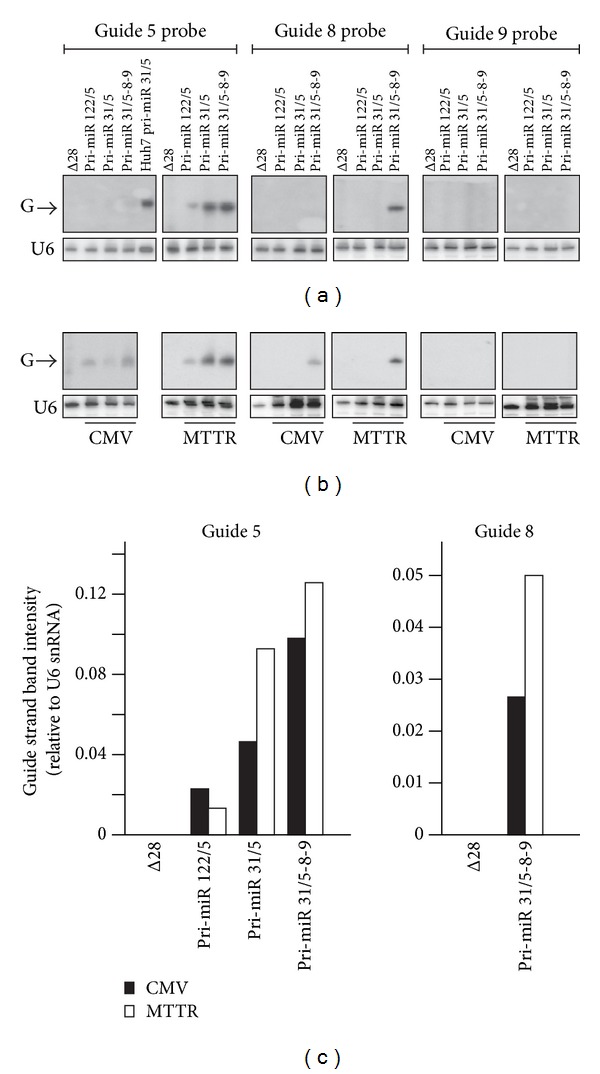
Anti-HBV pri-miR expression in livers of HBV transgenic mice following treatment with HD Ads expressing artificial mono- or trimeric pri-miRs under control of the CMV or MTTR promoters. Northern blot analyses of 30 *μ*g (a) and 60 *μ*g (b) of RNA isolated from mouse livers 1 week after infection with HD Ads. Blots hybridized with probes complementary to predicted guides 5, 8, or 9 were stripped and reprobed for U6 snRNA to confirm equal loading. (c) Quantification of indicated anti-HBV guide sequences generated from CMV and MTTR promoters was determined using a phosphorimager. Data were normalized to U6 snRNA expression and a representative example is shown.

To assess expression in vivo of anti-HBV pri-miRs derived from CMV and MTTR promoters, groups of mice were treated intravenously with 5 × 10^9^ HD Ad particles from each of the panel of HD Ad vectors. Seven days after vector administration, RNA was isolated from the livers and 30 *μ*g (Figure 4(a)) or 60 *μ*g (Figure 4(b)) was analyzed by northern blot hybridization. Detection of putative guide sequences correlated with analysis of RNA isolated from cultured liver-derived cells (Figure 3). Probing for guide 5 showed efficient expression and processing of anti-HBV pri-miRs in livers of animals that had been treated with mono- and trimeric HD Ads containing CMV or MTTR promoters. The guide 8 band was only detectable in RNA extracted from mice that received the HD Ads that contained the pri-miR-31/5-8-9 sequence. However the guide 9 sequence was not detectable in murine liver RNA samples and confirms inefficient processing of this component of the anti-HBV trimer. Production of guide strands from CMV- and MTTR-driven pri-miR cassettes was compared by determining guide strand band intensities following probe hybridization to 30 *μ*g (Figure 4(a)) or 60 *μ*g (Figure 4(b)) of RNA. Northern blot analysis of 30 *μ*g of RNA isolated from cells infected with HD Ad carrying cassettes containing the CMV promoter revealed a very faint band corresponding to the expected guides. Increasing the amount of RNA loaded onto the northern blots to 60 *μ*g improved detection of processed transcripts. Importantly, guide strands from RNA isolated from mice that had been treated with MTTR-containing plasmids were more easily measurable. Ratios of the guide strand to U6 snRNA loading control band intensities were used as an indicator of intrahepatocyte guide RNA concentrations (Figure 4(c)). This analysis revealed pri-miR expression and processing from the MTTR promoter was more efficient than that achieved with the CMV promoter.

To correlate detection of processed miR sequences with efficiency of hepatocyte transduction, we took advantage of HD Ad-mediated codelivery of the *β*-galactosidase expression cassette with the pri-miR expression cassettes. Liver sections stained for reporter gene activity confirmed previous observations that approximately 90% of murine hepatocytes had been transduced at two days after intravenous administration of the vector [24, 25]. However, a significant and rapid decline in the number of cells positive for *β*-galactosidase activity occurred thereafter (Figure 6(b)). By 6 weeks, reporter gene activity was barely detectable. Our previous reports indicate that the decline in cells positive for reporter gene activity is likely to be a result of an immune response to the cells expressing the reporter transgene [24].

### 3.5. Silencing of HBV Replication In Vivo by HD Ads Expressing Pri-miR Mimics from the MTTR Promoter

**Figure 5:**
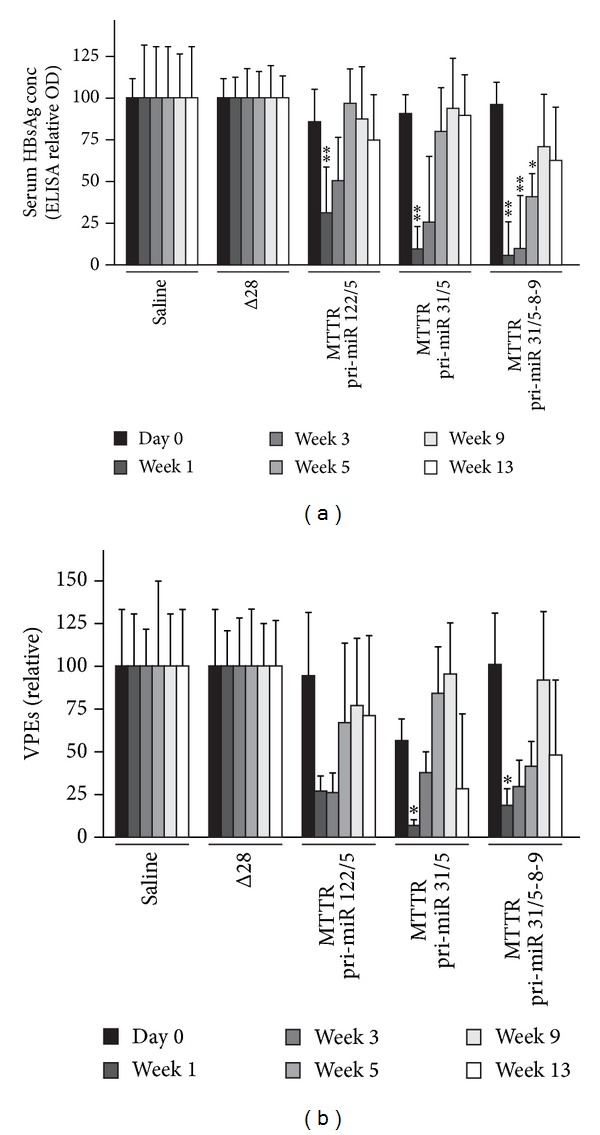
Silencing of HBV replication in vivo and reporter gene expression following administration of HD Ads expressing anti-HBV pri-miR mimics from the MTTR promoter. Mice were infected with a single dose of 5 × 10^9^ infectious HD Ad particles expressing pri-miR-122/5, pri-miR-31/5 and pri-miR-31/5-8-9. Animals injected with saline or HD Ad Δ28 served as negative controls. (a) Time course of serum HBsAg concentrations, as measured by ELISA, following HD Ad administration. (b) Circulating viral particles (VPEs) were measured using Q-PCR following injection of HBV transgenic mice with HD Ads. Data are expressed as means with SEMs from each group of six mice. The statistical significance was calculated using a pair-wise comparison according to the Student *t*-test. *P* values less than 0.05 (∗) or less than 0.01 (∗∗) were considered statistically significant.

To assess the silencing in vivo of HBV replication by HD Ads carrying the MTTR promoter-derived cassettes, both HBsAg levels and VPEs were determined in mice transduced with HD Ads. Six mice per group were injected with 5 × 10^9^ HD Ad infectious particles and blood samples collected over a period of 13 weeks. Control groups of animals were treated with saline or the HD Ad Δ28 empty vector. As with mice treated with the HD Ads expressing anti-HBV sequences from CMV promoter, a significant reduction in HBsAg levels was observed one week after vector administration (Figure 5(a)). HBsAg levels then increased significantly at 3 weeks after infection with HD Ads expressing the monomeric artificial pri-miRs from the MTTR promoter. However, HBsAg levels in mice treated with the cassette expressing the pri-miR trimer from the MTTR promoter remained significantly low at 3 and 5 weeks after infection (Figure 5(a)). Analysis of VPEs showed a similar trend in viral replication (Figure 5(b)).

### 3.6. HD Ad Vector Clearance and Toxicity in Mice

**Figure 6:**
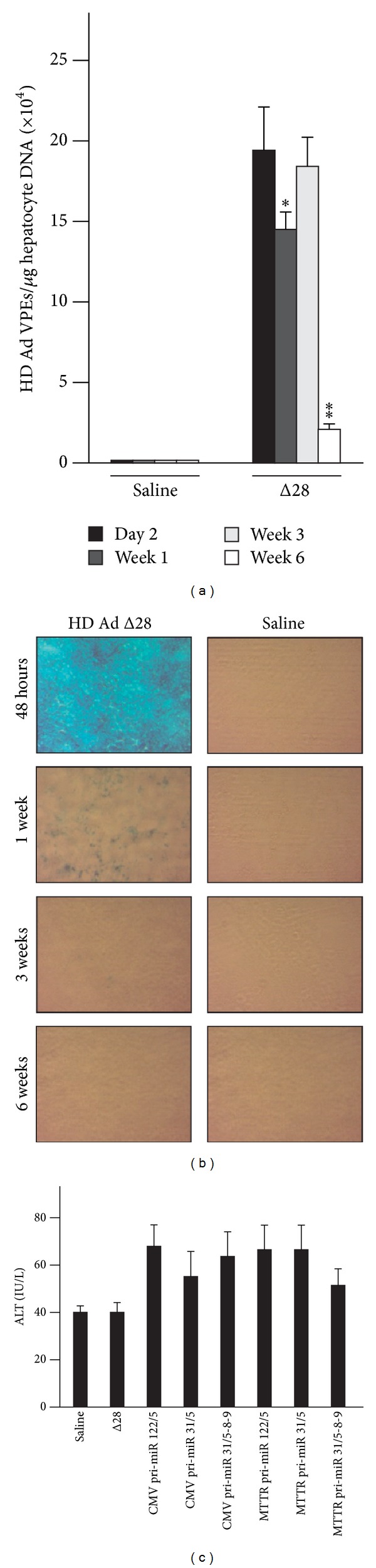
Intrahepatic HD Ad copy numbers and alanine transaminase activity following HD Ad administration to HBV transgenic mice. (a) Mice were injected intravenously with saline or a single dose of 5 × 10^9^ infectious viral particles. DNA isolated from mice livers harvested at 48 hours, 1 week, and 3 and 6 weeks after injection was subjected to Q-PCR analysis to determine viral particle equivalents (VPEs). Data are expressed as means (±SEM) from three mice. Statistically significant differences were determined by comparisons to values obtained at 2 days. Analyses were carried out using a pair-wise Student's *t*-test. *P* values less than 0.05 (∗) or less than 0.01 (∗∗) were considered statistically significant. (b) Histochemical detection of Beta-galactosidase activity in livers of mice treated with saline or HD Ad Δ28. Frozen liver sections were stained for reporter gene activity at 48 hours, 1 week, 3 weeks, and 6 weeks after saline or HD Ad Δ28 administration. (c) Serum ALT activity in HD Ad infected mice. Serum activity of ALT was measured 48 hours following injection with saline or 5 × 10^9^ infectious particles of the indicated HD As. Data is expressed as means (±SEM) from groups of six mice. The statistically significant differences were calculated as indicated above.

To determine whether vector clearance correlated with diminished efficacy against HBV replication, HD Ad genome copies were quantified in mouse livers. DNA isolated from liver samples collected at 48 hours, 1 week, and 3 and 6 weeks after HD Ad Δ28 infection were used to determine HD Ad vector genome copies in the liver. HD Ad Δ28 vector copies remained constant at approximately 2 × 10^5^ copies per *μ*g of hepatocyte DNA for the first three weeks after administration of HD Ads. Thereafter, the vector copies detectable in the livers diminished significantly at week 6 (Figure 6(a)), and this result correlated with disappearance of histochemically detectable Beta-galactosidase activity (Figure 6(b)). Q-PCR data from anti-HBV HD Ads showed similar trends of vector clearance (not shown) and indicated that the promoter sequences had little effect on viral vector clearance. Previously it has been reported that expression of anti-HBV shRNAs from a U6 Pol III promoter within adeno-associated vectors resulted in hepatotoxicity [9]. To assess toxicity of HD Ads in mice, ALT activity was measured 48 hours after injection of HD Ads. After 48 hours, ALT levels in control mice and animals injected with anti-HBV HD Ads were 51–69 U/L (Figure 6(c)). There were no statistically significant differences between the groups, which indicated that at an early time point, the anti-HBV HD Ads used in this study were not toxic to mice.

## 4. Discussion

Harnessing RNAi for the silencing of viral genes has demonstrated promising therapeutic potential. Several studies have shown that replication of viral pathogens can be inhibited with engineered mimics of the RNAi pathway. Chronic HBV infection is an important global public health problem and the inadequacies of currently available therapy have prompted investigation of the utility of RNAi-based treatment of the infection. Many studies have shown that HBV replication is susceptible to silencing by both synthetic and expressed RNAi activators. An advantage of using expressed RNAi activators to silence HBV gene expression is that it is possible to achieve more sustained silencing than with synthetic RNAi activators. This is critically important to effect durable silencing that is required to counter chronic HBV infection. Although there is enthusiasm for developing use of expressed RNAi activators as a therapeutic option for treating HBV persistence, important hurdles remain to be overcome before the approach becomes feasible. Particularly important are the controlled expression in target tissues, coupled to safe and efficient delivery of the expression cassettes to the liver.

Pol III promoters have been commonly used to express anti-HBV RNAi activator sequences [30, 37]. However, powerful constitutive activity of the U6 Pol III promoter may result in saturation of the RNAi pathway with resultant hepatotoxicity [9]. To address this concern, studies have aimed to use more versatile Pol II promoters to control production of antiviral sequences. Efficacy of shRNA mimics expressed from Pol II promoters is however variable and it appears that the flanking sequences naturally found in pri-miR sequences are important to facilitate expression and processing from Pol II promoters. As a result sequences that simulate the natural production of artificial pri-miR RNAi mimics have been engineered for incorporation into Pol II expression cassettes. In addition to compatibility with Pol II promoters, pri-miR sequences may be multimerized to enable simultaneous silencing at multiple viral targets. In this study we have demonstrated that the efficient liver-specific MTTR promoter may be used to express mono- and trimeric anti-HBV RNAi activators. Although the HBV genome is compact and not plastic, simultaneous targeting of three viral targets by the MTTR-controlled multimeric cassettes should prevent the emergence of viral escape mutants. Our observations demonstrated that the MTTR promoter functioned efficiently in vivo and was capable of significant silencing of HBV replication in a HBV transgenic mouse model.

We recently reported on efficacy in vivo of HD Ads that deliver anti-HBV pri-miR expression cassettes that are under control of the CMV promoter. Although effective against HBV in vivo [25], a concern of using the CMV promoter is that it is not specific to liver tissue and the transcription from this regulatory element is not sustained in hepatic tissue [35, 36]. The results reported here confirm that this is potentially problematic. Although concentrations of HD Ad DNA were maintained in murine liver tissue for at least 6 weeks, expression of the* lacZ *reporter gene, which was under control of the CMV transcriptional regulatory element, rapidly diminished 7 days after vector administration. A study by Löser and colleagues supports our observations [35]. In their investigation, expression of human low density lipoprotein (LDL) receptor from a CMV promoter was reduced after a week. However the protein was undetectable 4 weeks after vector administration despite the vector genome copy number remaining high at this time point. NF*κ*B deficiency and methylation of CpG sites within the CMV promoter sequence may be responsible for the attenuation of expression from this transcriptional regulatory element [35, 38].

Ads are highly efficient hepatotropic vectors that are well suited to delivery of RNAi expression cassettes that target HBV. However, the immunostimulatory effects of Ads [39–45] are concerning as they may cause toxicity, reduce efficiency of gene delivery, and limit transgene expression. Removal of ORFs to form HD Ads diminishes longer term humoral and cell mediated adaptive immunity to the vectors and has been useful to prolong transgene expression through [46]. Other modifications to HD Ads, such as conjugation of polymers, may also be used to diminish immunostimulatory effects. An additional important property of HD Ads is their capacity for accommodating large transgene sequences. Therefore as well as RNAi activating cassettes, other antiviral components may be incorporated into HD Ads to generate multifunctional vectors for HBV therapy. Recent advances in the use of transcription activator like effecter nucleases (TALENs) and zinc finger nucleases have been impressive [47]. These engineered gene-modifying enzymes may be particularly useful in combination with HBV-targeting RNAi activators to disable the stable HBV cccDNA minichromosome. Our data show that HD Ads have utility for hepatotropic delivery of a liver-specific expression cassette. Generating HD Ads that also include other antiviral sequences, a focus of our current research, should further add to their therapeutic utility.

## 5. Conclusions

The observation that the MTTR Pol II promoter achieves good expression of anti-HBV sequences is a useful property for the development of gene therapy requiring liver-specific expression of antiviral RNAi mimics. Transcription of HBV-targeting artificial mono- and trimeric pri-miRs under control of the MTTR element silenced HBV replication in vivo and generation of antiviral guides was more efficient than that achieved with the constitutively active CMV promoter. Moreover, expression of the antiviral sequences did not result in the hepatotoxicity that has been reported when U6 shRNA cassettes were delivered with adeno-associated viral vectors. The good delivery efficiency of recombinant HD Ads also reinforces the potential utility of these vectors. However diminishing immunostimulatory effects of the HD Ads, which may be achieved through removal of the codelivered* lacZ* and their modification with polymers, will need to be achieved before these vectors achieve clinical utility. HD Ads have the important property of being capable of carrying large transgene sequences. This will make it possible to augment their therapeutic efficacy by incorporating additional anti-HBV elements, such as those encoding engineered HBV DNA binding proteins. Advancing this approach, as well as attenuation of immunostimulatory effects of HD Ads are an active field of investigation in our laboratory.
